# Comparison of tramadol and lornoxicam for the prevention of postoperative catheter-related bladder discomfort: a randomized controlled trial

**DOI:** 10.1186/s13741-023-00317-z

**Published:** 2023-06-19

**Authors:** Xin Liao, Min Xie, Shuying Li, Xiaolan Yu

**Affiliations:** 1 Department of Operation Room, Key Laboratory of Birth Defects and Related Diseases of Women and Children, Ministry of Education, West China Second Hospital of Sichuan University, Sichuan Province, Chengdu, China; 2 Department of Anesthesiology, Key Laboratory of Birth Defects and Related Diseases of Women and Children, Ministry of Education, West China Second Hospital of Sichuan University, Sichuan Province, Chengdu, China

**Keywords:** Catheter-related bladder discomfort, Tramadol, Lornoxicam

## Abstract

**Background:**

Catheter-related bladder discomfort (CRBD is a painful complication of intraoperative urinary catheterization after anaesthesia. We conducted this study to compare the effect of tramadol and lornoxicam for the prevention of postoperative CRBD.

**Methods:**

One-hundred twenty patients (aged 18–60 years, ASA physical status 1–2, undergoing elective uterine surgery requiring intraoperative urinary catheterization were randomly divided into three groups with 40 patients in each group. Group T received 1.5 mg/kg tramadol, group L received 8-mg lornoxicam, and group C received normal saline. The study drugs were administered intravenously at the end of the surgery. The incidence and severity of CRBD were reported at 0, 1, 2, and 6 h after arrival at the postanaesthesia care unit (PACU).

**Results:**

The incidence of CRBD was significantly lower in groups T and L than in group C at 1, 2, and 6 h after surgery. The incidence of moderate to severe CRBD was also significantly lower in groups T and L than in group C at 0, 1, and 2 h after surgery. The severity of CRBD reported as mild, moderate, and severe was reduced in groups T and L compared with group C at most times after surgery. Group T had a higher incidence of nausea than group C, and there were no differences in dizziness, drowsiness, or vomit among the three groups.

**Conclusions:**

Tramadol and lornoxicam administered intravenously at the end of the surgery were both effective in preventing the incidence and severity of CRBD after uterine surgery. However, tramadol increased the incidence of nausea compared with saline, but there was no difference between tramadol and lornoxicam.

**Trial registration:**

ChiCTR2100052003. Registered on 12/10/2021.

## Background

Patients recovering from general anaesthesia after intraoperative insertion of urinary catheters often experience catheter-related bladder discomfort (CRBD). Postoperative CRBD is characterized by discomfort in the suprapubic region caused by catheter-related bladder irritation or is similar to the symptoms of overactive bladder such as urinary urgency and urinary frequency (Andersson 1993). CRBD is one of the most distressing complications that occur after surgery, which may increase postoperative pain and agitation and reduce the quality of recovery. The prevalence of CRBD ranges from 47 to 90%  (Andersson 1993; Binhas et al. 2011), so effective prevention and treatment are required due to its high incidence.

The pathophysiological mechanism of CRBD is thought to be caused by involuntary contraction of the bladder mediated by muscarinic stimulation, upregulation of C-afferent neuronal activity, and elevated PG synthesis stimulated by cyclooxygenase-2 (COX-2) (Andersson 1993; Bai et al. 2015; Igawa 2000; Groat et al. 2015; Yoshimura & Chancellor 2002). Various drugs have been used to prevent CRBD with varying degrees of success. However, the use of various drugs results in side effects such as dry mouth, sedation, nausea, and vomiting. Tramadol is a frequently used central-acting opioid analgesic that also has inhibitory effects on bladder M1 and M3 muscarinic receptors (Ergenoglu et al. 2012). It has been proven to be effective in preventing and treating CRBD (Bravo et al. 2017; Burimsittichai et al. 2017; Manandhar & Manandhar 2019). Lornoxicam is a COX inhibitor that is often used to manage postoperative pain. Some nonsteroidal anti-inflammatory drugs (NSAIDS), such as parecoxib, paracetamol, nefopam, and ketorolac, have been reported to effectively manage CRBD (Yoshimura & Chancellor 2002; Agarwal et al. 2008; Cheon et al. 2018; Park et al.; 2018; Jendoubi et al. 2018; Park et al.; 2019).

We supposed that lornoxicam might be as useful as tramadol for the prevention of postoperative CRBD. This study was designed to compare the efficacy of tramadol and lornoxicam administered at the end of uterine surgery on the incidence and severity of postoperative CRBD.

## Methods

This prospective randomized study was approved by the China Ethics Committee of Registering Clinical Trials and registered in the Chinese Clinical Trial Registry. Written informed consent was obtained from female patients with ASA classes 1–2, aged 18–60 years, who were scheduled to undergo elective uterine surgery requiring a urinary bladder catheter. Patients with a history of bladder outflow obstruction, urinary tract infection, overactive bladder, neurogenic bladder, chronic analgesic abuse, morbid obesity, psychiatric disease, and inability to communicate were excluded. Patients with intraoperative damage to the urinary tract or intestinal tract or massive haemorrhage were also excluded.

Patients were randomly divided into three equal groups with 40 patients in each group using computer-generated random numbers. The assignment was concealed in an envelope and opened immediately; the patient is entered in the operating room immediately by a nurse who was blinded to this study. Group T (tramadol group received 1.5 mg/kg tramadol, group L (lornoxicam group) received 8-mg lornoxicam, and group C received normal saline. The study drugs were administrated intravenously at the end of the surgery.

All patients received a consistent general anaesthesia without premedication. Anaesthesia was induced with midazolam 2 mg, propofol 1.5–2 mg/kg, and sufentanil 0.3–0.4 μg/kg, and tracheal intubation was facilitated by rocuronium 0.6–0.9 mg/kg. Urinary catheterization was performed using a 16-F Foley urinary catheter with 10-ml distilled water inflating the balloon after induction of anaesthesia. Anaesthesia was maintained with sevoflurane and intermittent dosages of sufentanil and vecuronium when required. At the end of surgery, postoperative nausea and vomiting were prevented with 3-mg granisetron, while neuromuscular blockade was reversed with neostigmine when needed. All patients received patient-controlled intravenous analgesia (PCIA) with sufentanil (0.5 μg/ml) and tramadol (4 mg/ml). After tracheal extubation, patients were transferred to the postanaesthesia care unit (PACU) for further recovery.

Bladder discomfort was assessed at 0, 1, 2, and 6 h after arrival to the PACU with the help of a blinded nurse. Patients were instructed how to differentiate CRBD from preoperative pain. The severity of CRBD was reported as none when there was no complaint of CRBD when asked, as mild when recorded by patients only when asked, as moderate when reported by patients spontaneously (without being asked and not accompanied by any behavioural response), and as severe when reported by patients with spontaneous behavioural responses (flailing limbs, strong vocal response, and attempt to remove the catheter). Associated adverse effects, such as nausea, vomiting, drowsiness, and dizziness, were also recorded.

The sample size was calculated based on the previous studies, which showed that the incidence of CRBD among the three groups ranged from 0.3 to 0.7 (Manandhar & Manandhar 2019; Li et al. 2019). Power analysis with *a* = 0.05 and *b* = 0.10 indicated that 37 patients were needed in each group. To accommodate for dropouts, if any, 40 patients were enrolled in each group. The incidence and severity of CRBD and adverse effects were reported as percentage frequencies and were analysed by the chi-square test. Demographic data, surgery and anaesthesia duration time, sufentanil dosage, and urine output were evaluated by one-way analysis of variance (ANOVA). Statistical analysis was analysed using SPSS 20.

## Results

**Fig. 1:**
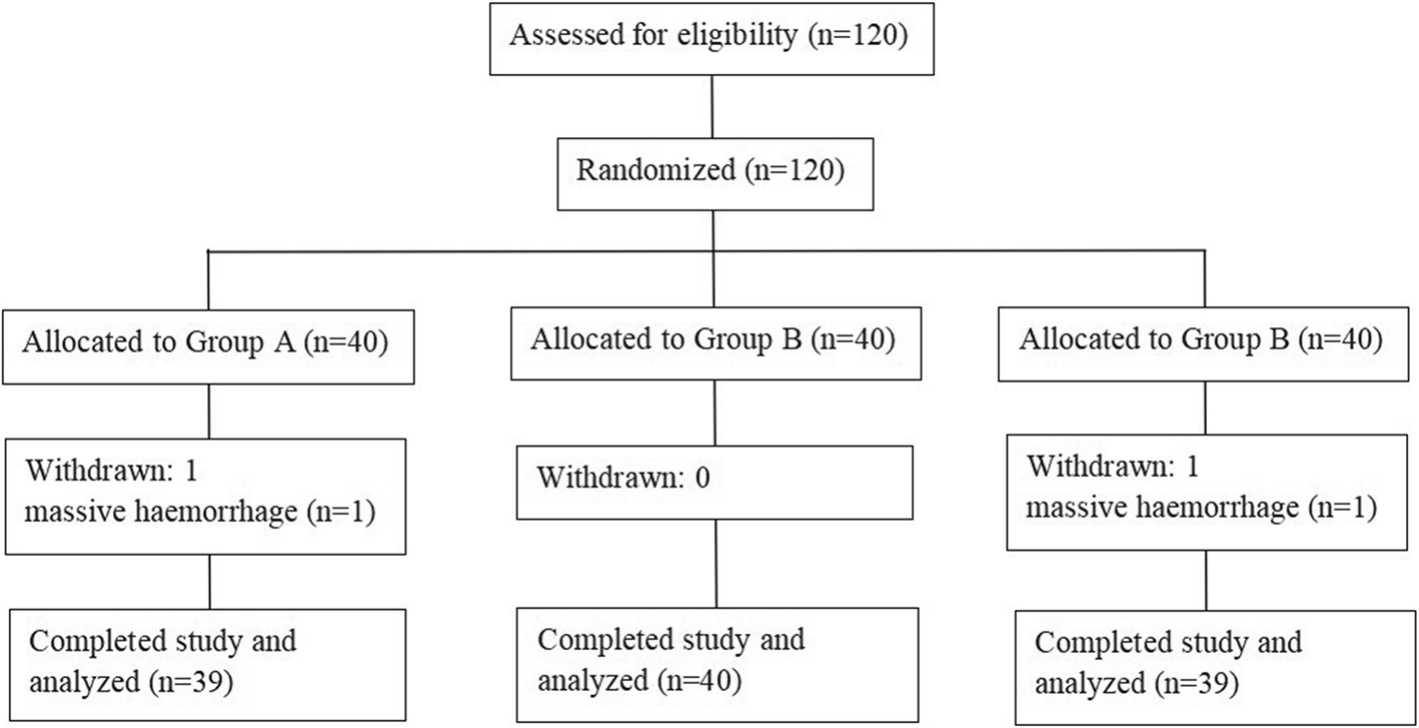
CONSORT flow diagram

A total of 120 patients were enrolled in this study. Two patients were excluded for massive haemorrhage (Fig. 1). There were no significant differences among the three groups with regard to patient characteristics, surgery and anaesthesia duration time, sufentanil dosage, or urine output (Table 1).

**Table 1:** Patient’s characteristics and clinical data among the three groups

	Group T	Group L	Group C	*P*
Number of patients (*n*)	39	40	39	
Age (year)	48.0 ± 8.3	46.1 ± 8.1	48.3 ± 7.6	0.410
Height (cm)	157.5 ± 5.6	158.5 ± 3.9	157.0 ± 5.7	0.39
Weight (kg)	57.5 ± 6.4	57.9 ± 8.2	57.6 ± 9.4	0.966
Laparoscopic/open surgery (*n*)	5/34	6/34	6/33	
Surgery duration time (min)	135.1 ± 57.4	138.6 ± 50.5	129.5 ± 51.5	0.749
Anaesthesia duration time (min)	160.6 ± 58.8	162.0 ± 49.3	154.1 ± 51.0	0.782
Sufentanil dosage (µg)	27.1 ± 5.1	25.3 ± 5.2	25.5 ± 5.1	0.255
Urine output (ml)	218.2 ± 147.2	233.5 ± 129.8	252.6 ± 180.6	0.615

**Fig. 2:**
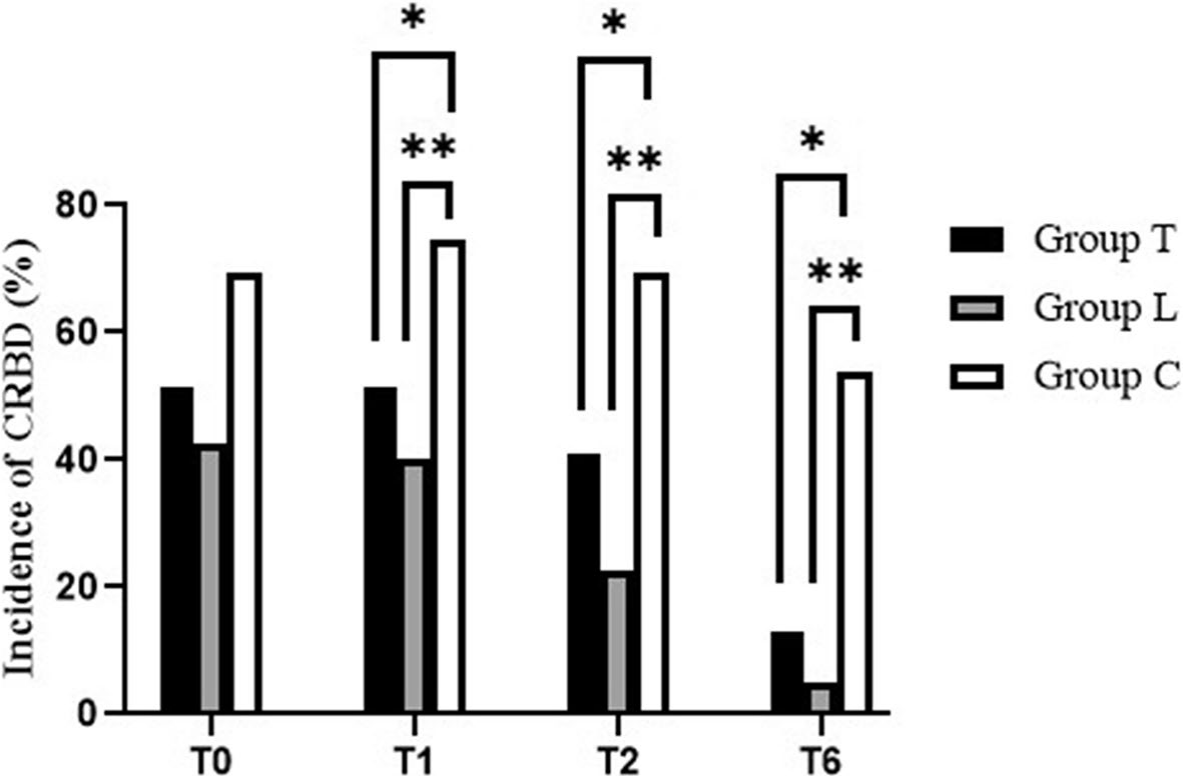
The incidence of CRBD among the three groups. *P < 0.05 for comparison between group T vs group C; **P < 0.05 for comparison between group L vs group C

**Fig. 3:**
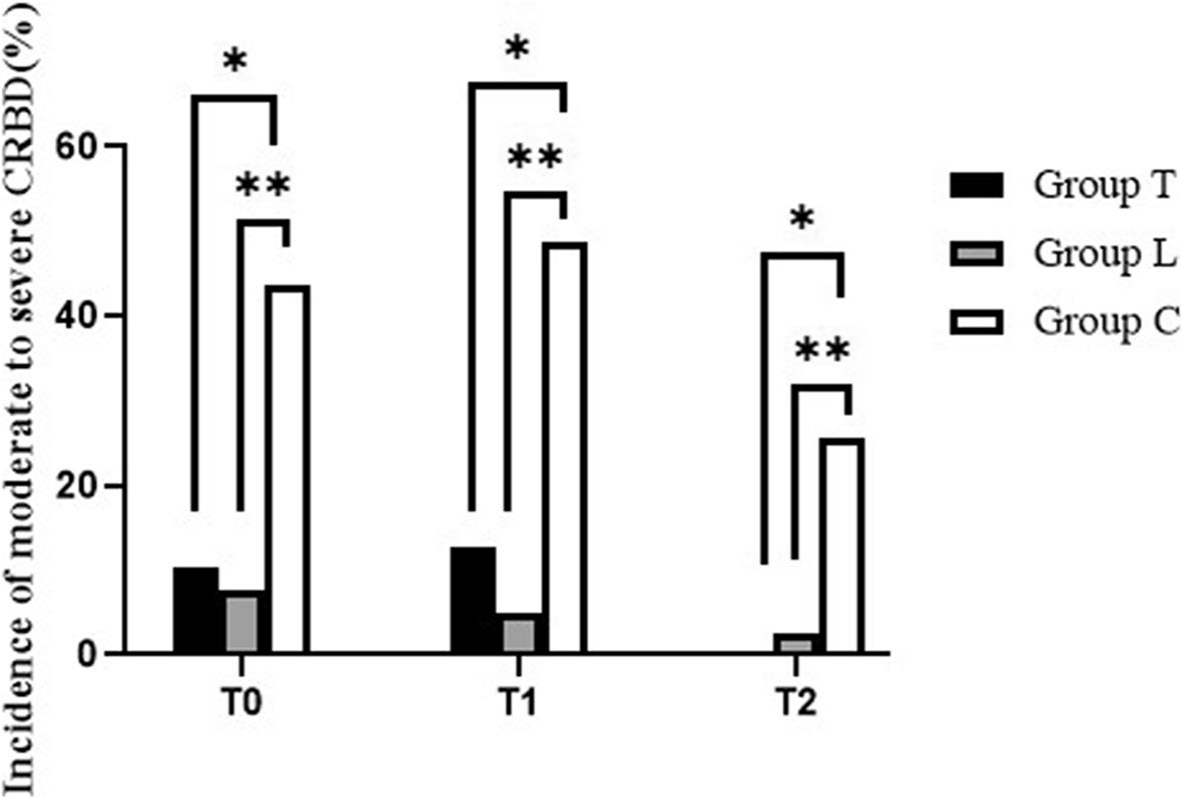
The incidence of CRBD above mild among the three groups. *P < 0.05 for comparison between group T vs group C; **P < 0.05 for comparison between group L vs group C

**Fig. 4:**
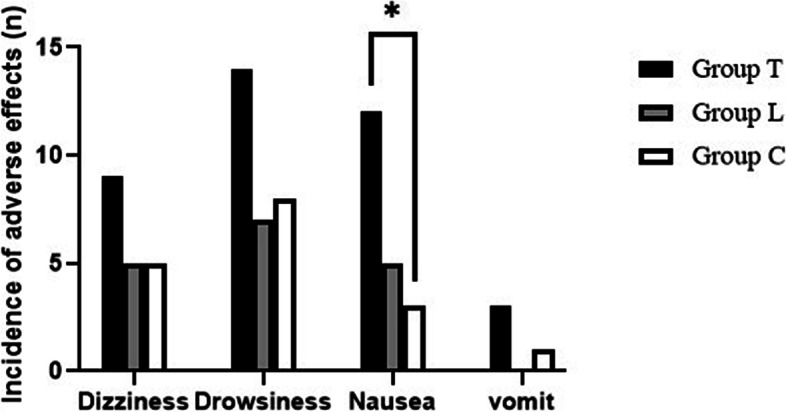
Adverse effects among the three groups. *P < 0.05 for comparison between group T vs group C

The incidence of CRBD was significantly lower in groups T and L than in group C at 1, 2, and 6 h after surgery (Fig. 2). The incidence of moderate to severe CRBD was also significantly lower in groups T and L than in group C at 0, 1, and 2 h after surgery (Fig. 3). The severity of CRBD reported as mild, moderate, and severe was reduced in groups T and L compared with group C at most times after surgery (Table 2. Group T had a higher incidence of nausea than group C, and there were no differences in dizziness, drowsiness, or vomit among the three groups (Fig. 4).

**Table 2:** Incidence and severity of CRBD presented as numbers (*n*)

Time Group	T0			T1			T2			T6		
	T	L	C	T	L	C	T	L	C	T	L	C
Incidence	20	17	27	20*	16**	29	16*	9**	27	5*	2**	21
Severity												
No	19	23	12	19	24**	10	23*	31**	12	34*	38**	18
Mild	16*	14	5	15	14	10	16	8	17	5*	2**	21
Moderate	4*	3**	16	5*	2**	18	0*	1**	10	0	0	0
Severe	0*	0**	6	0	0	1	0	0	0	0	0	0

^*^P < 0.05 for comparison between group T vs group C

^*^^*^P < 0.05 for comparison between group L vs group C

## Discussion

In this study, we found that tramadol administered intravenously at the end of the surgery was as effective as lornoxicam in preventing the incidence and severity of CRBD after uterine surgery. However, tramadol increased the incidence of nausea.

Patients with urinary catheters awakening from anaesthesia often complain of postoperative CRBD. It is widely recognized that postoperative CRBD is a risk factor for postoperative emergence agitation. It has been reported that operation type is one of the risk factors for CRBD, such as urological surgeries and lower abdominal surgeries having a higher incidence of CRBD (Lim & Yoon 2017; Li et al. 2014). Our previous study has found that uterine-related surgery, age, and additional analgesics were the independent predictor of CRBD after gynaecological surgery, while type of surgery (laparoscopic surgery, open abdominal surgery, cervical conization, and pelvic surgery); occasion of catheterization (before anaesthesia, after anaesthesia); intraoperative atropine or postoperative neostigmine and atropine usage; and postoperative pain were not the independent predictor (Li et al. 2020). In this study, patients undergoing uterine surgery had a high incidence of CRBD of up to 74%, and the incidence of moderate to severe CRBD which usually needed treatment was up to 48.7%.

Bladder involuntary contraction mediated by muscarinic receptors is the main mechanism of CRBD. Among the five types of muscarinic receptors, the *M*_3_ receptor is related to direct contraction, and the *M*_2_ receptor is responsible for indirect contraction of the bladder (Binhas et al. 2011). Tramadol is a central-acting opioid analgesic that is frequently used for postoperative pain relief. It can inhibit noradrenaline and serotonin reuptake and has an inhibitory effect on *M*_1_ and *M*_3_ muscarinic receptors. Some studies reported that intravenous administration of tramadol 1–1.5 mg/kg intravenous administered was effective in preventing the incidence and severity of CRBD after some surgeries (such as upper urinary tract surgery, percutaneous nephrolithotomy, or laparoscopic surgery) (Bravo et al. 2017; Burimsittichai et al. 2017; Manandhar and Manandhar 2019). Our previous study reported that tramadol 1.5 mg/kg was effective in treating CRBD after elective gynaecological surgery (Li et al. 2018). Our recent systematic review evaluated the different interventions for preventing of CRBD in different surgeries. Three RCTs were included in tramadol group and found that tramadol showed a significant efficacy to prevent CRBD at 0, 1, 2, and 6 h postoperatively (Li et al. 2022).

Our study found that lornoxicam was as effective as tramadol in preventing the incidence and severity of CRBD, and this result was consistent with previous studies on NSAIDS. NSAIDS, such as parecoxib, paracetamol, nefopam, and ketorolac, have been found to effectively prevent the incidence and severity of CRBD. In a preclinical study, lornoxicam was found to be the most potent balanced inhibitor of COX-1/-2. The equipotent COX isoenzyme inhibition of lornoxicam was complemented by a marked inhibition of IL-6 production and inducible nitric oxide (iNOS)-derived nitric oxide (NO) formation. The study also found that the lornoxicam has dose dependently inhibited the NO formation, whereas piroxicam, diclofenac, ibuprofen, ketorolac, and naproxen were markedly less (Berg et al. 1999). It has been reported that patients with overactive bladder and lower urinary tract obstruction have a higher level of PGE_2_, and that prostaglandins (PGs) may play an important role in the development of these diseases (Kim et al. 2006, 2005; Aoki et al. 2009). PG is synthesized by COX in the bladder, and its synthesis is caused by various physiological stimuli (Andersson 2010; Andersson and Arner 2004). Lornoxicam is a COX inhibitor that may act by inhibiting the capacity of PG synthesis to inhibit CRBD. Previous study found lornoxicam (i.v. 8 mg at the end of the operation and at 12 h postoperatively) and tramadol (i.v. 1 mg/kg at the end of the operation and every 6 h up to 24 h postoperatively) were provided rapid and effective analgesia; there were no differences between the two drugs as regard to the pain score (Mentes et al. 2009). Lornoxicam 8 mg was a common clinical dose, so in this study, we chose lornoxicam 8 mg compared with tramadol 1.5 mg/kg (Yücel et al. 2016a, b; Işik et al. 2009; Yücel et al. 2016a, b).

Regarding the adverse effects of the study drugs, our study found that tramadol increased the incidence of nausea compared with saline, but there were no differences between tramadol and lornoxicam. There were no differences in dizziness, drowsiness, or vomiting. Our recent systematic review also evaluated the adverse effects of all different drugs and found that tramadol significantly increases the incidence of PONV (Li et al. 2022). For overall consideration, lornoxicam is a better option than tramadol for the management of CRBD.

There were some limitations in this study. First, we did not evaluate the efficacy of tramadol and lornoxicam on all types of surgeries, and different types of surgeries might have some associated interference. Moreover, all the enrolled patients were women, so male sex might also have interfered. Furthermore, the observation time was only limited to only 6 h postoperatively, and we failed to report CRBD beyond 6 h. However, our previous research was found that the incidence of moderate to severe CRBD was 0 (Li et al. 2019).

Tramadol and lornoxicam administered intravenously at the end of the surgery were both effective in preventing the incidence and severity of CRBD after uterine surgery. However, tramadol increased the incidence of nausea.

## Data Availability

The datasets are not publicly available but available from the corresponding author on reasonable request.

## Abbreviations

CRBD: Catheter-related bladder discomfort
COX-2: Cyclooxygenase-2
NSAIDS: Nonsteroidal anti-inflammatory drugs
PACU: Postanaesthesia care unit
ANOVA: One-way analysis of variance
PGs: Prostaglandins
